# Unknown rheumatic cardiac disease as cause of acute onset post-partum dyspnea: a case report

**DOI:** 10.1186/s12884-023-05809-w

**Published:** 2023-07-03

**Authors:** António De Pinho, Andreia Mota De Sousa, Anabela Melo, Anabela Ferreira

**Affiliations:** 1 Obstetrics and Gynecology Department - Tâmega E Sousa Hospital Center, Penafiel - Porto, Avenida do Hospital Padre Américo 210, Penafiel, 4564-007 Portugal; 2 Biomedicine Department - Faculty of Medicine, University of Porto, Porto, Portugal; 3 Pediatrics and Gynecology/Obstetrics Department - Faculty of Medicine, University of Porto, Porto, Portugal

**Keywords:** Dyspnea, Postpartum period, Pregnancy, Pulmonary edema, Rheumatic heart disease

## Abstract

**Background:**

Acute post-partum dyspnea configures an obstetric challenge with multiple differential diagnosis.

**Case presentation:**

We present a case of a previous healthy woman with preeclampsia who developed severe dyspnea 30 h after delivery. She complained of cough, orthopnea, and bilateral lower extremities oedema. She denied headaches, blurry vision, nausea, vomiting, fever or chills. Auscultation revealed a diastolic murmur, and was compatible with pulmonary oedema. A timely bedside echocardiogram showed moderate dilated left atrium with severe mitral insufficiency suggestive of an unknown rheumatic disease. She was managed with noninvasive ventilation, loop diuretics, vasodilators, thromboprophylaxis, head-end elevation, and fluid restriction with progressive improving.

**Conclusions:**

Hemodynamic changes in pregnant patients with previously silent cardiac disease may pose a challenge and cause post-partum dyspnea. This scenario requires a timely and multidisciplinary approach.

## Background

The investigation of an acute onset dyspnea in the postpartum period should include a complete evaluation for causes, such as previously silent cardiac diseases [1]. Several physiological hemodynamic changes take place in pregnancy. On average, the cardiac output increases, especially by the time of delivery and in the early puerperium. This fact coupled with an increase in the heart rate may pose a very vulnerable period for the pregnant cardiovascular patient [2].

## Case presentation

A foreign African black 28-year-old pregnant woman (3 previous gestations, 1 live birth, 1 abortion) with no previous chronic comorbidities was diagnosed with mild preeclampsia (de novo hypertension plus proteinuria) at 36 weeks of gestation. The patient had regular antenatal visits, no specific signs or symptoms related with previous cardiovascular disease, and the pregnancy evolved uneventfully until the 36^th^ week. Her obstetric history revealed a previous diagnosis of preeclampsia and preterm labor, with absence of a post-partum revision consultation to seek for underneath pathologies. 

Labor was induced at 37 weeks with an uncomplicated eutocic delivery. Approximately 30 h after delivery, she presented chief complaint of increasing shortness of breath, and chest pressure. Patient also reported cough, orthopnea, and bilateral lower extremities oedema. She denied headaches, blurry vision, nausea, vomiting, fever or chills. During observation, she was in severe distress, restless, agitated, and using her accessory muscles of respiration. Vitals were recorded, which revealed normal temperature, tachycardia (120 bpm), tachypnea (28 cpm), and mild hypertension (143/86 mmHg). She had a peripheral oxygen saturation of 83%. Cardiac auscultation revealed tachycardia and a diastolic murmur more perceptible in the mitral area, and respiratory auscultation revealed fine crackles heard bilaterally with decreased basilar breathing sounds. Bilateral pitting pedal oedema was noted. Therefore, a multidisciplinary team approach with Obstetrics, Internal Medicine and Cardiology team was implemented.

Arterial blood gas analysis on Venturi mask 28% revealed pH of 7.46, carbon dioxide tension 33 mmHg, arterial oxygen tension 51 mmHg, bicarbonate 23.5 mmol/L, and arterial oxygen saturation of 88%. Routine blood investigations (full blood count, liver and kidney function tests, prothrombin time/international normalized ratio, serum electrolytes, and urine analysis) were drawn. This pannel showed no abnormalities, besides mild leukocytosis. Brain natriuretic peptide (BNP) was raised—1011 pg/mL. Cardiac enzymes were negative. Serial 12 leads electrocardiogram showed sinus tachycardia, with T-wave inversion in V1-V3. SARS-CoV2 Real Time-PCR was negative. A 2D bedside echocardiogram was performed revealing moderate dilated left atrium with severe mitral insufficiency suggestive of rheumatic disease, normal systolic function, and no other alterations.

After initial stabilization, patient was admitted to the ICU for further supervision. She was managed with noninvasive ventilation, loop diuretics, vasodilators (nifedipine), thromboprophylaxis, head-end elevation, and fluid restriction with progressive improving. Four days after delivery, the patient was stable and was discharged.

A Cardiology and Obstetrics post-partum revision consultation was conducted, 6–8 weeks after delivery. By that time, the patient was asymptomatic without the need for any anti-hypertensive or diuretic drug. An autoimmunity panel was conducted, in the context of preeclampsia at young age, which resulted negative. A new echocardiogram was conducted showing an improvement of parameters with mild mitral disease with a rheumatic pattern, and no other relevant changes.

Patient was also referred for dental evaluation and informed of the impact of her rheumatic cardiac disease in the current pregnancy and the need for adequate follow-up in case of eventual future pregnancies.

## Discussion and conclusions

**Fig. 1:**
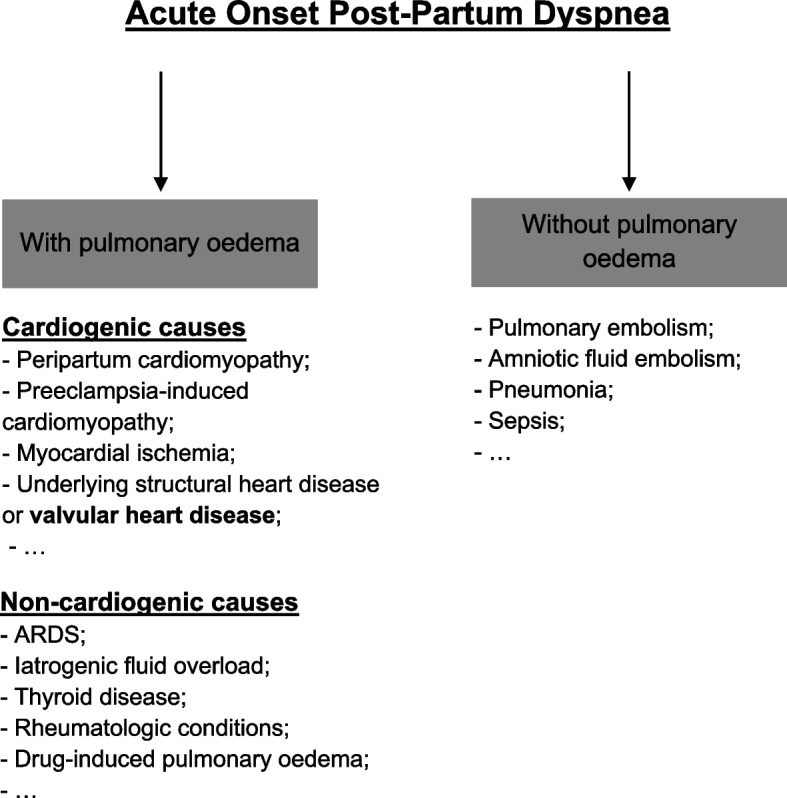
Legend: main causes of acute onset post-partum dyspnea

Many conditions can result in acute onset post-partum dyspnea [3] (Fig. 1). These conditions include pathologies not associated with pulmonary oedema, such as pulmonary embolism, amniotic fluid embolism, pneumonia (e.g. bacterial, COVID-19), sepsis, and pathologies associated with pulmonary oedema [4]. In the last group, the causes may be cardiogenic, including peripartum cardiomyopathy, preeclampsia-induced cardiomyopathy, myocardial ischemia, and underlying structural heart disease or valvular heart disease (as in this clinical case). Examples of noncardiogenic pulmonary oedema are acute respiratory distress syndrome (ARDS), iatrogenic fluid overload (e.g., during labor), thyroid disease, rheumatologic conditions, and drug-induced pulmonary oedema (e.g., by oxytocin).

In European countries, rheumatic valvular disease is a rare yet important cause of post-partum dyspnea with cardiogenic pulmonary oedema, but this condition remains prevalent in women from low resource-settings [2]. De novo diagnosis should be considered especially in patients originally from endemic areas. Rheumatic cardiac disease may be silent and present for the first time in the peripartum period [5]. In the presented case, the demographic context, symptoms, a complete physical examination, increase of BNP, and doppler echocardiography findings were the key for diagnosis. Echocardiography is one of the most widely available imaging modalities and plays an important role in diagnosis, and assessment of the severity of valvular pathology, and cardiac function. Besides transvalvular gradients (velocity-derived measurements) alone, other markers of severity as left atrium size should also be considered in pregnancy [6].

In patients with previous diagnosis of rheumatic cardiac disease, preconception counselling is essential, although less than 50% of women with cardiac disease in the United Kingdom are adequately assessed by a Cardio-Obstetrics team before pregnancy [7]. Usually, mitral regurgitation configures a class III (high risk) condition in pregnancy, according to the modified World Health Organization classification [2]. In the setting of a severe rheumatic mitral regurgitation or with left ventricle dysfunction in preconception, a definitive cardiac intervention should be considered.

During pregnancy, diuresis and fluid management should be managed carefully, and the use of angiotensin-converting enzyme inhibitors is contraindicated. If progressing or refractory symptoms with medical therapy, surgery may be needed in very few cases during gestation. In rheumatic cardiac disease patients’ vaginal delivery ± instrumentation is usually indicated, and c-section is mainly reserved for obstetric reasons, or in selected cases with severe stenotic lesions. A valve specific follow-up plan should be based on clinical and echocardiogram evaluations 6–8 weeks after delivery [2]. 

## Data Availability

The datasets used and analyzed during the current study is available from the corresponding author on reasonable request.
